# Cardioprotection provided by Echinatin against ischemia/reperfusion in isolated rat hearts

**DOI:** 10.1186/s12872-016-0294-3

**Published:** 2016-05-31

**Authors:** Xing-han Tian, Chao-liang Liu, Hai-Li Jiang, Yan Zhang, Ji-chun Han, Ju Liu, Meng Chen

**Affiliations:** Intensive Care Unit, Yantai Yuhuangding Hospital of Laishan branch, Yantai, China; Cardiovascular Department of Affiliated Hospital of JiNing Medical University, Jining, China; Department of Oncology, Shuguang Hospital, Shanghai University of Traditional Chinese Medicine, Shanghai, China; Department of Internal medicine, Qihe people’s hospital, Dezhou, China; Department of Internal medicine, Yantai Yuhuangding Hospital of Laishan branch, Yantai, China; Medical Research Center, Shandong Provincial Qianfoshan Hospital, Shandong University, Jinan, China

**Keywords:** Echinatin, Cardioprotection, Ischemia/reperfusion, Anti-inflammatory, Antioxidant

## Abstract

**Background:**

This study evaluated the protective effect of Echinatin against myocardial ischemia/reperfusion (I/R) injury in rats.

**Methods:**

The effect of Echinatin on cardiac function in rats subjected to I/R was demonstrated through improved Langendorff retrograde perfusion technology. Adult Sprague–Dawley rats were randomly divided into five groups, and myocardial infarct size was macroscopically estimated through 2,3,5-triphenyltetrazolium chloride staining. The coronary effluent was analyzed for the release of lactate dehydrogenase (LDH) and creatine kinase (CK) to assess the degree of cardiac injury. The concentrations of malondialdehyde (MDA), interleukin-6 (IL-6), and tumor necrosis factor-α (TNF-α) were determined along with superoxide dismutase (SOD) activity using ELISA. Finally, cardiomyocyte apoptosis analysis was conducted with POD, an in situ cell death detection kit.

**Results:**

Echinatin (0.5 and 2.5 μg/mL) pretreatment enhanced the maximum up/down rate of the left ventricular pressure (±dp/dtmax), improved the heart rate, increased the left ventricular developed pressure (LVDP), enhanced the coronary flow, and reduced the CK and LDH levels in the coronary flow of the treated group compared with the I/R group. Echinatin limited the contents of CK and LDH, improved the LVDP, reduced the contents of MDA, IL-6, and TNF-α, and increased the SOD activity. The infarct size and cell apoptosis in the hearts of the rats in the Echinatin-treated group were smaller and lower, respectively, than those in the hearts of the rats in the I/R control group.

**Conclusion:**

Echinatin exerts a protective effect against I/R-induced myocardial injury on hearts. This effect may be attributed to the antioxidant and anti-inflammatory activities of this compound.

## Background

Cardiovascular diseases (CVDs) are the main cause of morbidity and mortality all over the world [1]. Theoretically, restoring blood supply to the ischemic myocardium can reduce myocardial injury; however, reperfusion can aggravate the myocardial damage caused by ischemia/reperfusion (I/R) injury [2]. A patient’s condition can be seriously exacerbated by such injury, and thus the prevention of I/R is important in alleviating ischemic heart disease.

I/R injury is an intricate process that implicates many mechanisms, such as oxidative damage [3]. Several studies revealed that the oxidative stress caused by reactive oxygen species (ROS) plays a significant role in such injury and impairs cardiac function. I/R causes an imbalance between antioxidants and toxic free radicals, and this imbalance increases the susceptibility of tissues to oxidative damage through lipid peroxidation and protein oxidation [4, 5]. Apoptosis and inflammation also play important roles in the progression of I/R injury. Several works reported that antiapoptosis and anti-inflammation protect the heart from I/R injury and provide additional evidence suggesting that both mechanisms are factors in this injury [6, 7]. Therefore, the antioxidants and anti-inflammatory drugs obtained from plants represent a logical therapeutic strategy to treat I/R injury.

**Fig. 1:**
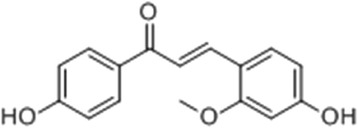
Chemical structure of Echinatin

The licorice (liquorice) plant has a long and storied history of use in both Eastern and Western cultures. Some licorice extracts exhibit unique antioxidant properties and anti-inflammatory activities; in particular, licochalcones B to D exert antioxidative and cardioprotective effects [8–10]. Echinatin (molecular formula: C_16_H_14_O_4_; Fig. 1) is a licorice extract that displays antioxidant properties and anti-inflammatory activities [11]. Therefore, the present study evaluates the mechanisms and effects of Echinatin in rats with I/R.

## Methods

### Test compounds, chemicals, and reagents

Echinatin (purity ≥ 98 %) was purchased from Chengdu Must Biotechnology Co., Ltd. (Chengdu, China). 1,1,3,3-Tetramethoxypropane was obtained from Fluka Chemical Co. (Ronkonkoma, NY). 2,3,5-Triphenyltetrazolium chloride (TTC) and both oxidized and reduced glutathiones were purchased from Sigma Chemical Co. (St. Louis, MO). The other chemicals and reagents used were of analytical grade.

### Animals and experimental groups

Adult Sprague–Dawley rats (Jinan Jinfeng Experimental Animal Breeding Co., Ltd.; License Number: SCXK(lu) 2014–0006) weighing 250–300 g were housed in the animal facility of the Yantai Yuhuangding Hospital Experimental Animal Center in accordance with the institutional guidelines. The rats were randomly divided into five groups: control (sham), I/R, and Echinatin (0.1, 0.5, and 2.5 μg/mL) treatment groups. Hearts in the control group were stabilized for 30 min and perfused for 65 min, there is not zero-flow global ischemia in whole process. The hearts of the rats in the I/R group were subjected to 20 min of zero-flow global ischemia and 45 min of reperfusion after stabilization. The hearts of the rats in the treatment groups were stabilized for 30 min and then treated with a Krebs–Henseleit (K–H) buffer solution containing Echinatin (0.1, 0.5, and 2.5 μg/mL). Global ischemia and reperfusion were established for 45 min.

### I/R model establishment

Sprague Dawley rats (250–300 g) were anesthetized with an intraperitoneal injection of 60 mM chloral hydrate (0.35 g/kg). To prevent coagulation of the blood, 250 U/kg of heparin were administered intraperitoneally. The heart was then excised quickly by thoracic surgery and immediately mounted on Langendorff’s apparatus. The hearts were immersed in ice-cold Krebs-Henseleit buffer (118 mM NaCl, 1.2 mM KH_2_PO_4_, 4.7 mM KCl, 1.7 mM CaCl_2_, 1.2 mM MgSO_4_, 20 mM sodium acetate and 10 mM glucose, pH 7.4), equilibrated with a gas mixture comprised of 95 % O_2_/5 % CO_2_ at 37 °C, and then maintained in a water-jacketed organ chamber at 37 °C. A water-filled latex balloon coupled to a pressure transducer (Statham) was inserted into the left ventricular cavity via the left auricle for recording pressure.

This study evaluated cardiac function by monitoring hemodynamic parameters, which are important indices of cardiac function. The Echinatin dose applied in the experiments was determined through preliminary experiments, wherein Echinatin was tested at doses of 0.1, 0.5, and 2.5 μg/mL. The following functional parameters were continuously monitored with a computer-based data acquisition system (PC PowerLab with Chart 5, 4S AD Instruments): left ventricular developed pressure (LVDP), maximum rise/down velocity of left intraventricular pressure (dp/dtmax and dp/dtmin) and coronary flow (CF). The heart effluents were collected at 1 min intervals to determine the coronary flow (CF). The recovery of LVDP, dp/dtmax, dp/dtmin and CF were expressed as the percent of 1 min before ischemia.

### Measurement of cellular injury

The levels of lactate dehydrogenase (LDH) and creatine kinase (CK) released were measured to evaluate the occurrence of necrotic cell death. After the experiment, these levels in the perfusate were spectrophotometrically determined with cytotoxicity detection kits for LDH and CK (Nanjing Jiancheng Biological Product, Nanjing, China).

### Evaluation of myocardial infarct size

The artery was occluded for 20 min and then reperfused for 45 min prior to terminating the experiment based on successful ischemia and reperfusion methods previously used in the same experimental model. To assess tissue death, the hearts were removed and washed in phosphate-buffered saline, frozen, stored at −20 °C for 30 min, and then sliced into 1 mm-thick sections perpendicularly along the long axis from the apex to the base. The slices were incubated in 1 % TTC of the pH 7.4 buffer at 37 °C for 10–15 min, fixed in 10 % formaldehyde solution, and then photographed with a digital camera to distinguish the red-stained viable tissues from the white-unstained necrotic tissues. The red- and white-stained areas were measured using an Image-Pro Plus 7.0 instrument (Media Cybernetics, Wyoming, USA). The percentage of infarction size was calculated with the following equation: %Infarct volume = Infarct volume/Total volume of slice × 100.

### Assay of oxidative stress and inflammation

At the end of the experiment, the hearts were harvested and maintained at −70 °C for subsequent analysis. The frozen ventricles were crushed to powder by a liquid nitrogen-chilled tissue pulverizer. The weighed amount of frozen tissues was homogenized in the appropriate buffer using a microcentrifuge tube homogenizer for tissue analysis.

Superoxide dismutase (SOD) activity and the levels of malondialdehyde (MDA), tumor necrosis factor-α (TNF-α), and interleukin-6 (IL-6) were spectrophotometrically analyzed through ELISA (Tsz Biosciences, Greater Boston, USA) according to the manufacturer’s instructions.

### Terminal deoxynucleotidyl transfer-mediated dUTP nick end-labeling (TUNEL) staining

We conducted TUNEL staining using POD, an in situ cell death detection kit (Roche, Germany), according to the manufacturer’s instructions. Following deparaffinization and rehydration, the sections were treated with 10 mM protease K for 15 min. The slides were immersed in a TUNEL reaction mixture for 60 min at 37 °C in a humidified atmosphere in the dark. The slides were incubated in Converter-POD for 30 min to show blue nuclear staining and then analyzed via optical microscopy. The TUNEL index (%) was computed by dividing the ratio of the number of TUNEL-positive cells by the total number of cells; this index was considered in evaluating the apoptosis index of TUNEL-stained heart tissues. Eight randomly selected areas of TUNEL-stained slices were counted for each sample, and the average value was calculated.

### Statistical analysis

The data were presented as mean ± SD from at least three independent experiments and were assessed through Student’s *t*-test. Statistical significance was set to *p* < 0.05, and the statistical analysis was conducted with SPSS (IBM SPASS, International Business Machines Corporation, USA).

## Results

### Echinatin enhances the recovery of I/R-altered cardiac function

**Fig. 2:**
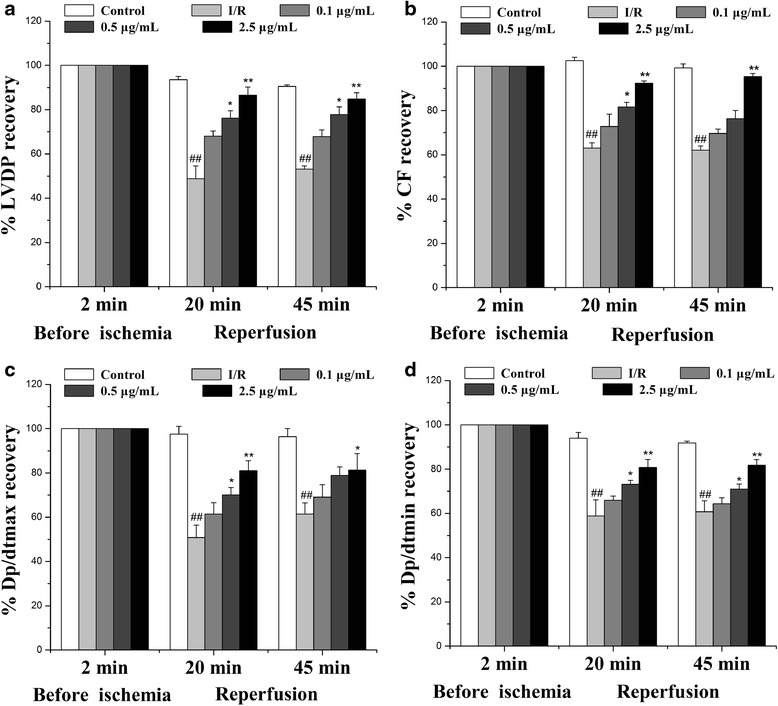
Effect of Echinatin on the cardiac function of rats subjected to I/R. Effect of Echinatin on (**a**) LVDP, (**b**) CF, (**c**) dp/dtmax, and (**d**) dp/dtmin in rat hearts. (Values are expressed as mean ± SD, *n* = 6). ^*##*^ *P < 0.01* compared with the control group; *P < 0.05, **P < 0.01 compared with the I/R group

Hemodynamic parameters were continuously monitored using a computer-based data acquisition system. The effects Echinatin of treatment on LVDP, ±dp/dtmax and CF are shown in Fig. 2. The functional recovery of the Echinatin-treated hearts was significantly greater than that of the unprotected I/R hearts during reperfusion. In particular, Echinatin doses of 0.5 and 2.5 μg/mL improved LVDP and -d*p*/d*t*_max_ considerably (*p < 0.05, **p < 0.01, respectively); in addition, a 2.5 μg/mL dose significantly enhanced + d*p*/d*t*_max_ (*p < 0.05) and CF (**p < 0.01).

### Echinatin attenuated the release of I/R-induced enzymes in rat hearts

**Fig. 3:**
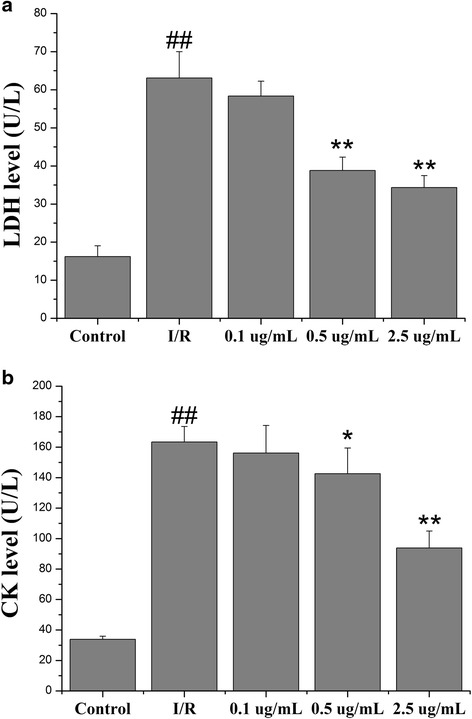
Effect of Echinatin on (**a**) LDH and (**b**) CK levels in coronary effluent during I/R injury (Values are expressed as mean ± SD, *n* = 6). ^*##*^ *P < 0.01* compared with the control group; *P < 0.05, **P < 0.01 compared with the I/R group

This study measured the release of LDH and CK to evaluate the degree of myocardial injury. This method was used in previous studies to determine the occurrence of necrotic cell death prior to ischemia. After 20 min of ischemia that was followed by 30 min of reperfusion, CK and LDH leakage was notably greater in the I/R group than in the control group (Fig. 3). Echinatin pretreatment at doses of 0.5 and 2.5 μg/mL significantly reduced the I/R-induced increase in the LDH and CK releases in the rat hearts (**p < 0.01, *p < 0.05, and **p < 0.01).

### Echinatin reduced the I/R-induced infarct size

**Fig. 4:**
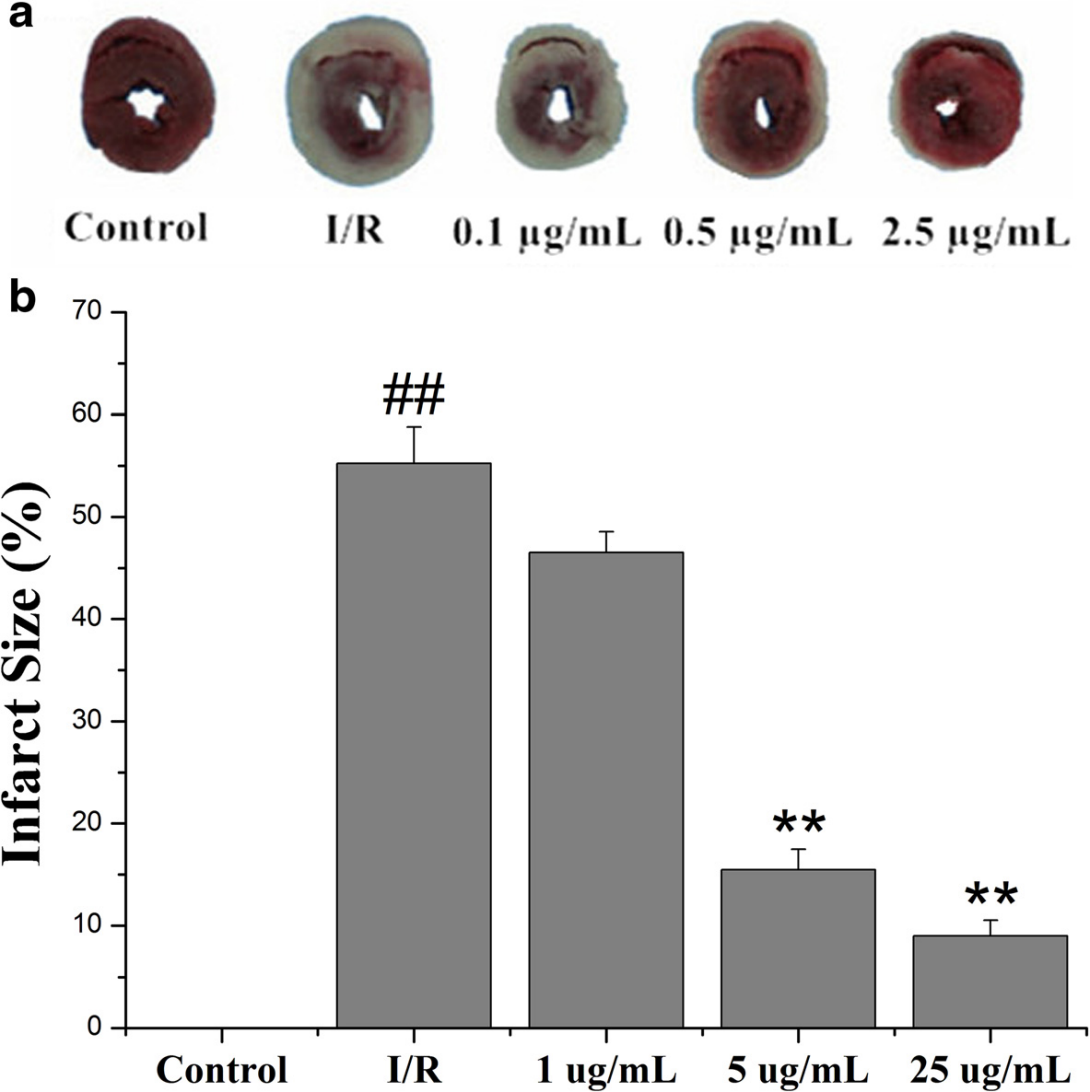
Effect of Echinatin on the reduction of I/R-induced infarct size. (**a**) The myocardial infarct size; (**b**) The infarct size shown as percentage. (Values are expressed as mean ± SD, *n* = 6). ^*##*^ *P < 0.01* compared with the control group; **P < 0.01 compared with the I/R group

As shown in Fig. 4, myocardial infarct size can indicate myocardial injury. The infarct area increased significantly in the rat hearts in the I/R group (55.24 % ± 3.56 %). By contrast, 0.5 and 2.5 μg/mL of Echinatin treatment reduced I/R-induced myocardial infarct size by 15.49 % ± 1.98 % and 8.97 % ± 1.51 %, respectively (**p < 0.01).

### Echinatin alleviated the oxidative stress induced by I/R injury on myocardial tissues

**Fig. 5:**
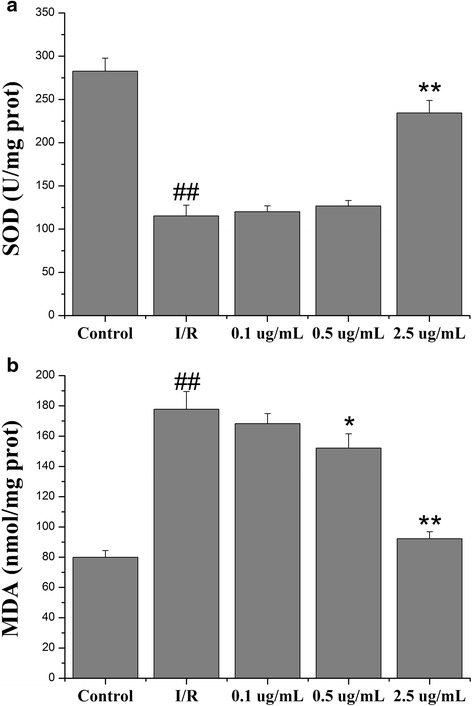
Effects of Echinatin on the (**a**) SOD activity and (**b**) MDA level of isolated rat hearts subjected to I/R (Values are expressed as mean ± SD, *n* = 6). ^*##*^ *P < 0.01* compared with the control group; *P < 0.05, **P < 0.01 compared with the I/R group

ROS generation is a major factor in I/R injury. To identify the possible mechanisms of Echinatin on cardioprotection, the MDA level and SOD activity were determined in the myocardial tissue. These indicators were also calculated in such tissues to identify the possible mechanisms underlying the cardioprotective effects of Echinatin. Pretreatment with 2.5 μg/mL of Echinatin significantly increased the SOD activity (Fig. 5a) (**p < 0.01)*,* whereas pretreatment with 0.5 and 2.5 μg/mL Echinatin doses considerably lowered the MDA level (Fig. 5b) (*p < 0.05 and **p < 0.01, respectively).

### Echinatin attenuated the myocardial tissue inflammation induced by I/R injury

**Fig. 6:**
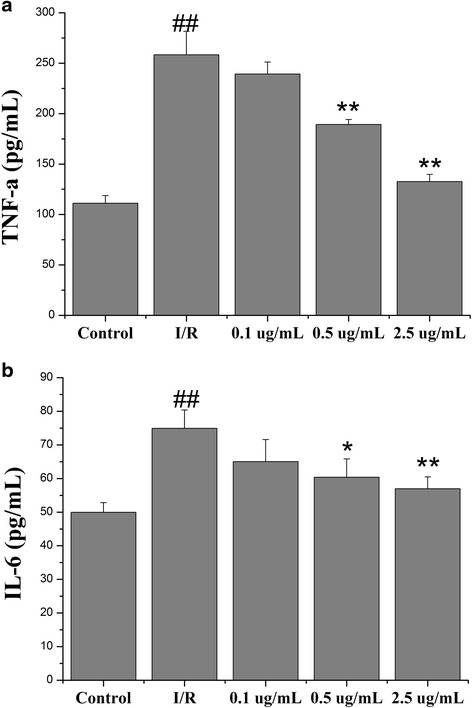
Effect of Echinatin on (**a**) TNF-α and (**b**) IL-6 levels in rats subjected to I/R (Values are expressed as mean ± SD, *n* = 6). ^*##*^ *P < 0.01* compared with the control group; *P < 0.05, **P < 0.01 compared with the I/R group

Inflammation is an important mechanism underlying myocardial I/R injury. The presence of the inflammatory cytokines (e.g., IL-6 and TNF-α) associated with I/R injury was determined in myocardial tissues to identify the possible mechanisms behind the cardioprotective activity of Echinatin. Moreover, the IL-6 and TNF-α activities were measured. The TNF-α content in the groups pretreated with Echinatin at 0.5 (189.31 ± 4.82 pg/mL) and 2.5 μg/mL (132.72 ± 7.04 pg/mL) was significantly lower (**p < 0.01) than that in the I/R group (258.35 ± 23.18 pg/mL) (Fig. 6a). The IL-6 activity decreased from 74.92 ± 5.46 pg/mL in the I/R group to 56.96 ± 3.54 pg/mL in the group pretreated with 2.5 μg/mL Echinatin (**p < 0.01) (Fig. 6b).

### Echinatin minimized the cardiomyocyte apoptosis induced by I/R injury

**Fig. 7:**
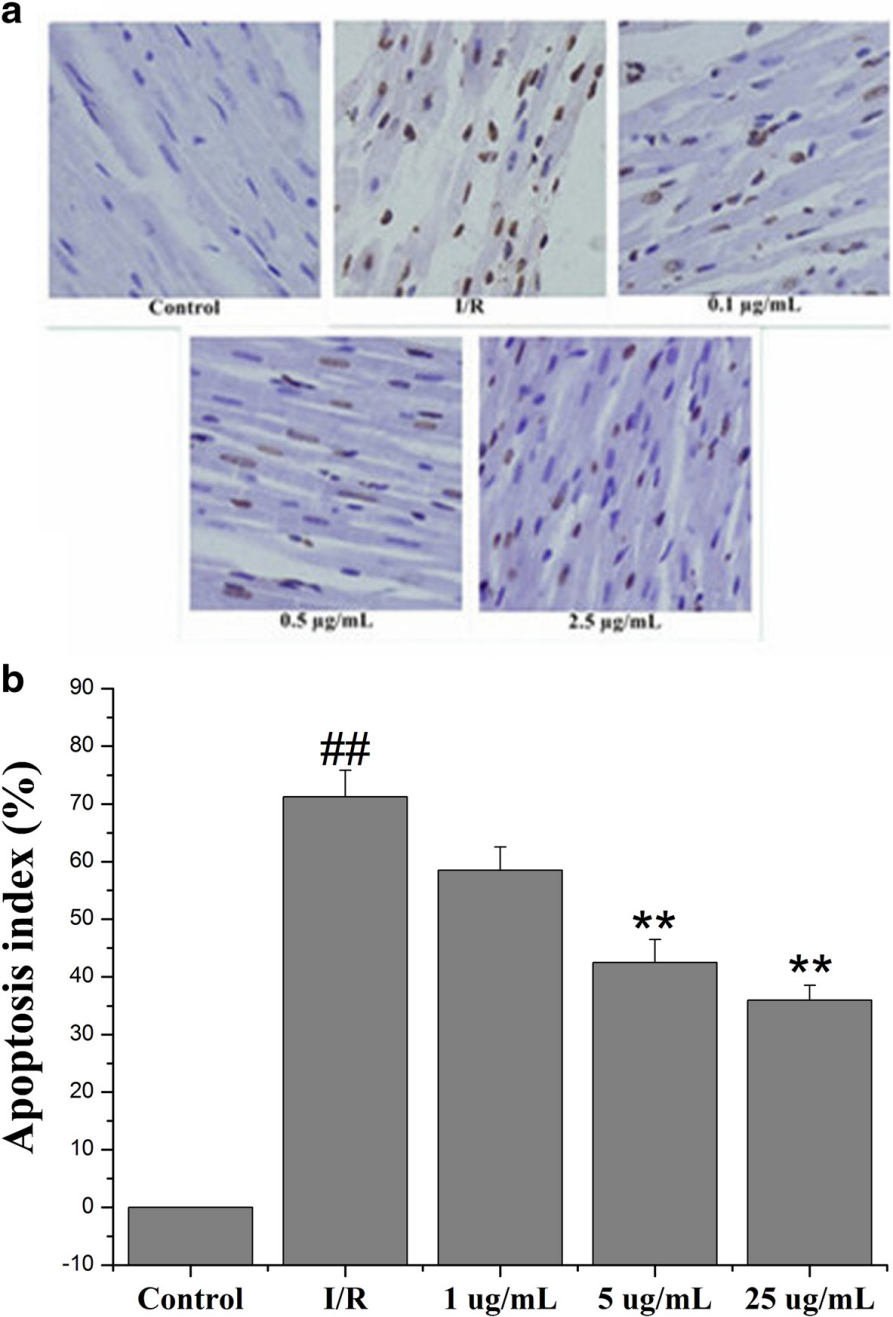
Effects of Echinatin on the suppression of cardiomyocyte apoptosis (×200). The brown nucleus indicates the apoptotic cardiomyocyte nucleus. (**a**) The TUNEL staining; (**b**) Percentage of apoptotic cells. (Values are expressed as mean ± SD, *n* = 6). ^*##*^ *P < 0.01* compared with the control group; **P < 0.01 compared with the I/R group

This section discusses the results of the myocardial ischemic reperfusion in the event of cardiomyocyte apoptosis, which was observed in this study via TUNEL staining. As illustrated in Fig. 7, this staining highlighted the lack of apoptosis in the control group under optical microscopy. The number of apoptotic cells increased considerably in the I/R group but visibly declined in the groups pretreated with Echinatin at doses of 0.5 and 2.5 μg/mL (**p < 0.01).

## Discussion

We investigated the effects of Echinatin on cardiac function, myocardial enzymes, inflammatory factors, and cardiomyocyte apoptosis in the I/R model of an isolated rat heart. We then confirmed that Echinatin improves cardiac function recovery, reduces intracellular oxidation status, and inhibits I/R-induced cardiomyocyte apoptosis.

Myocardial I/R results in heart dysfunction [8, 9]. We observed significant myocardial dysfunction, including changes in hemodynamic parameters (LVDP, ±dp/dtmax and CF), release of enzymes (CK and LDH), and induced myocardial infarct after myocardial I/R. These phenomena agree with the results of numerous reports, indicating that reperfusion is a key initiator of myocardial dysfunction associated with I/R injury. Echinatin significantly improved the recovery of I/R-altered hemodynamic parameters (LVDP, ±dp/dtmax and CF), decreased I/R-induced enzyme release (CK and LDH) and reduced infarct size.

Researchers have reached a consensus that oxidative stress can trigger significant myocardial injury after I/R through certain pathways [12, 13]. During reperfusion, excessive amounts of ROS are generated by myocardial cell mitochondria and xanthine oxidase pathways; these amounts are beyond the endogenous capability of cells. MDA (a critical component of the NADPH oxidase) is produced through lipid peroxidation, myocardial enzymes are released, structural proteins are attacked, and cell structures are destroyed [14, 15]. As oxidative stress is a crucial event in I/R injury, oxidative stress inhibition is considered a viable approach to treat I/R-induced cardiac injury. In this experiment, Echinatin treatment significantly reduced the MDA content in I/R rats and improved the I/R-induced deterioration of total antioxidant capacity (SOD activity). Thus, Echinatin plays a cardioprotective role by regulating oxidative stress.

Inflammation, which plays an important role in many disease states, is associated with enhanced expression of adhesive molecules in the vasculature, resulting in the infiltration of larger populations of neutrophils and monocytes/macrophages. The release of pro-inflammatory cytokines from these activated leukocytes can then in turn cause tissue damage [16]. Several lines of evidence suggest that I/R induces vigorous inflammatory reactions, such as a significant increase in the levels of IL-6 and TNF-α in the myocardial tissue. These proinflammatory cytokines from these activated leukocytes can then in turn cause tissue damage [17, 18]. Along these lines, inflammation plays a key role in cardiac I/R injury, and the deleterious events that follow these events include an increased release of proinflammatory mediators (e.g., TNF-α, CRP and IL-6). To investigate the relationship between the anti-inflammatory and cardioprotective effects of Echinatin, an experiment was conducted to determine whether or not this extract affected the I/R-induced changes in IL-6 and TNF-α. The findings showed that I/R increased the production of these cytokines, whereas Echinatin treatment reduced their concentrations. Overall, this extract may inhibit myocardial I/R injury through anti-inflammatory activities.

Such injury results in heart dysfunction and cardiomyocyte apoptosis [19, 20]. In fact, several studies have clarified the deleterious effects of I/R injury [21]. In our experiments, Echinatin treatment improved cardiac function recovery and significantly reduced the apoptotic index of rats after I/R. Thus, this compound can protect the heart by minimizing apoptosis.

## Conclusions

Echinatin facilitated cardioprotection against myocardial I/R injury, and this capability may be attributed to its antioxidant, anti-inflammatory, and anti-apoptotic activities. Thus, we hypothesize that Echinatin deserves additional experimental and clinical research in the cardiovascular milieu.

## Abbreviations

±dp/dtmax, maximum up/down rate of the left ventricular pressure; CF, coronary flow; CK, creatine kinase; I/R, ischemia/reperfusion; IL-6, interleukin-6; LDH, lactate dehydrogenase; LVDP, left ventricular developed pressure; LVEDP, left ventricular end-diastolic pressure; MDA, malondialdehyde; SOD, superoxide dismutase; TNF-α, tumor necrosis factor-α; TTC, 2,3,5-Triphenyltetrazolium chloride
